# Coping strategies and quality of life in caregivers of dependent elderly relatives

**DOI:** 10.1186/s12955-017-0634-8

**Published:** 2017-04-14

**Authors:** Margarita Rodríguez-Pérez, Ana Abreu-Sánchez, María Jesús Rojas-Ocaña, Rafael del-Pino-Casado

**Affiliations:** 1 School of Health Sciences, Department of Nursing, University of Huelva, Huelva, Spain; 2 School of Health Sciences, Department of Nursing, University of Jaén, Jaén, Spain

**Keywords:** Caregivers, Elderly, Coping, Quality of life, Nursing

## Abstract

**Background:**

Despite the importance of coping in caregiving, there are few studies on the relationship between coping and quality of life in caregivers of the frail dependent elderly. Thus, this study aims to analyze the relationship between coping strategies and quality of life dimensions in primary caregivers of dependent elderly relatives.

**Methods:**

A cross-sectional study was conducted from 86 caregivers. Predictive variables were coping strategies (problem-focused, emotion-focused, socially-supported, and dysfunctional); dependent variables were quality of life dimensions (psychological, physical, relational, and environmental); and potential confounding variables were age, gender, perceived health and burden of caregiver, and functional capacity of care receiver. Correlation coefficients were calculated and multiple linear regression analysis was performed.

**Results:**

After controlling for potential confounders, dysfunctional coping was related to worse quality of life in the psychological dimension, while emotion-focused and socially-supported coping were related to superior psychological and environmental dimensions of quality of life. The physical and relational dimensions of quality of life were not related to coping strategies.

**Conclusions:**

1) it is important to consider coping strategies in the assessment of primary caregivers of dependent elderly relatives; 2) the quality of life of caregivers is related to their coping strategies, 3) their quality of life can be worsened by avoidance-type coping, and 4) their quality of life can be improved by active emotion-focused coping and socially-supported coping.

## Introduction

Care of the dependent elderly by relatives is increasingly frequent with the aging of populations and represents the main care resource [1]. It can have a negative impact on the health and wellbeing of the caregiver, which has been attributed to stress [2]. The family member taking on this responsibility must adapt to a new challenge and, as affirmed by Lazarus & Folkman [3], stress can result from situations perceived by the individual as potentially overwhelming and a threat to their well-being and quality of life. The burden of caring for a dependent person can impair the physical and mental health of the caregiver [4]. However, the response of caregivers can be positive or negative depending on various factors [5], such as the coping strategies of caregivers [6].

In their multidimensional Stress Process Model, Pearlin, Mullan, Semple, & Skaff [7] categorized four types of variables that affect the well-being and quality of life of the caregivers: contextual variables, primary objective stressors, secondary stressors, and modulating variables. In this model, coping strategies have a modulating function that allows the recording of different individual responses to the same care situation.

## Background

### Coping

Coping, defined as cognitive and behavioral efforts to manage demands perceived as taxing or exceeding the resources of an individual [3], has been classified according to various criteria [8]. Thus, authors have distinguished between emotion-focused and problem-focused coping [3] or between active or approach and passive or avoidance coping [9]. Problem-focused coping is oriented toward resolving challenges, while emotion-focused coping is geared to managing emotions. Approach coping includes attempts to reappraise, modify, and solve problems, while avoidance coping is related to attempts to avoid problems and engage in indirect attempts to reduce distress. Both approach and avoidance coping can be classified as either behavioral or cognitive [9].

In stressful situations, the objective of a coping strategy is to solve the problem or regulate the emotional response [10]. The effectiveness of different strategies is controversial [11, 12]. According to Penley et al. [10], both problem-focused and emotion-focused coping, although conceptually distinct, can reduce psychological distress and are both used in most stressful episodes. Bass [13] and Folkman [14] pointed out that coping strategies alone are not effective against caregiver stress, and that the response will depend on the nature of stressors. However, various authors (e.g.: [15]) have observed that the management of stress by coping increases the quality of life.

### Quality of life

The World Health Organization (WHO) has defined the quality of life as: “an individuals’ perception of their position in life in the context of the culture and value systems in which they live and in relation to their goals, expectations, standards, and concerns. It is a broad ranging concept affected in a complex way by the person’s physical health, psychological state, level of independence, social relationships, personal beliefs, and their relationship to salient features of their environment” [16].

In conceptual models used in caregiving research, caregivers’ quality of life has been considered as a final outcome in the caregiving situation, with several factors related to this quality of life, such as coping [17, 18]. Various researchers have investigated the quality of life of informal caregivers [19], but there have been few studies on the relationship between coping and quality of life in informal care and even fewer in informal care of the frail dependent elderly. A search of PubMed for “caregivers[mh] AND Coping[tiab] AND quality of life[ti]”, with no time limit, only yielded two articles on the dependent elderly [20, 21] and four articles on dependent adults including the elderly [19, 22–24]. Moreover, the results of studies on coping and quality of life in informal care have been inconsistent. Thus, avoidance coping strategies have been associated with a worse quality of life by some authors [23] and an improved quality of life by others [20]. Likewise, active-type strategies have also been associated with a worse quality of life by some authors [24] and an improved quality of life by others [22].

A limitation of many studies on the relationship between coping and quality of life is the failure to control for potential confounders (e.g.: [23]). Various characteristics of caregivers have been associated with an improved quality of life, including older age [25], female sex [26], lesser perceived care burden [27], and better perceived health status [28]. A superior functional status of the care receiver has also been associated with a better quality of life of the caregiver [22].

Greater understanding of the relationship between coping and quality of life is required to support and promote the development of interventions to improve the lives of caregivers [29].

With this background, the objective of the present study was to analyze the relationship between coping strategies and quality of life dimensions in the primary caregivers of dependent elderly relatives, controlling for age, sex, perceived health and burden of the caregiver, and the functional capacity of the care receiver.

## Methods

### Design, setting and sample

A descriptive cross-sectional study was conducted in primary caregivers of dependent elderly relatives attended in an urban health center in Huelva (Southern Spain). This center serves a population of around 20,000 inhabitants with a mixed socioeconomic level.

A convenience sample was recruited from caregivers who met inclusion criteria. These inclusion criteria were: being a primary caregiver, age >18 yrs, caregiver for > 3 months, no receipt of remuneration for the care, the ability to read and write, and care of a dependent elderly relative aged > 65 yrs dependent in at least one basic activity of daily living (BADL) according to the Barthel Index [30]. The primary caregiver was defined as the person who takes the main responsibility in caring for the care recipient, provides the largest amount of daily care for this care recipient, and maintains liaison with the formal health providers.

Out of the 176 primary caregivers of dependent elderly attended in the health center, study eligibility criteria were met by 108. After the exclusion of 10 caregivers who were hospitalized and 12 who refused participation, the remaining 86 caregivers gave their written consent and were enrolled in the study. Hospitalization leads to not participation because interviews were developed at home and primary caregivers were with the care recipient in the hospital. Data were gathered between June and December 2010.

The sample size estimation was based on the recommendation by Nunnally and Bernstein [31] for multiple linear regression of a minimum of 10 subjects per independent variable. Given that eight independent variables (four predictive variables and four control variables) were considered in the present study, a minimum sample size of 80 units was required. According to Cohen [32], this sample size permits a regression analysis that offers 84% statistical power with 95% confidence interval for four predictive variables that explain at least 12% of the variance in the dependent variable and four control variables that explain at least 15% of this variance (calculated with PASS 11).

### Measures

#### Coping (independent variable)

Coping was evaluated with the Spanish version by Crespo and Cruzado [33] of the COPE Inventory [34]. It includes 60 items with responses on a Likert scale from 1 (= I normally do not do this at all) to 5 (= I normally do this a lot). A dispositional version of the questionnaire was used in the present study (how the subject normally copes with stress situations). Following the proposal by Litman [35], items were classified into the following third-order dimensions based on the second-order dimensions described by Carver et al. [34]: 1) problem-focused coping (active coping centered on the problem): active coping, planning, and suppression of competing activities; 2) emotion-focused coping (active coping centered on emotions): restraint coping, reinterpretation, acceptance, humor, and religion; 3) avoidance coping (passive or avoidance coping): denial, behavioral disengagement, emotional disengagement, and substance; 4) socially-supported coping (active coping focused on seeking social support): the search for social support for instrumental and emotional reasons.

In the present study, the internal consistency (Chronbach’s alpha) results for these dimensions were 0.74 for problem-focused coping, 0.70 for emotion-focused coping, 0.67 for avoidancetype coping, and 0.78 for socially-supported coping.

#### Quality of life (dependent variable)

Quality of life was evaluated using the WHO QOL-BREF scale [36] based on WHOQOL-100. The WHOQOL-BREF scale was validated in Spain using a sample of 558 outpatients, psychiatric patients, and individuals from the general population along with their caregivers; they reported a correlation of around 0.9 between WHOWOL-BREF scores and WHOQOL-100 scores and an internal consistency (Cronbach’s alpha) score for the WHOQOL-BREF of > 0.7 in all domains [37].

The scale includes 26 items on physical and psychological status, social relationships, and environmental setting, with responses on a Likert-type scale from 1 to 5. Items are classified into four domains: psychological, physical, social relational, and environmental. The psychological domain addresses positive feelings, spirituality/religion, thought, learning, memory, concentration, body image/physical appearance, self-esteem, and negative feelings. The physical domain includes perception of pain/discomfort, dependence on medication, level of fatigue/energy, capacity for mobility, quality of sleep/rest, and capacities for daily activities and work. The relational domain considers personal relationships, sexual activity, and social support, while the environmental domain includes physical safety/protection, physical environment, economic resources, opportunity/capacity to acquire information, household, and availability of healthcare and transport. For each item, a higher score indicates superior quality of life. The scale do not provide a unique score but one for each dimension.

In the present study, the internal consistency (Cronbach’s alpha) results were 0.79 for psychological domain, 0.80 for physical domain, 0.70 for relational domain, and 0.71 for environmental domain.

#### Control variables

The potential confounders considered were: age (years), sex, perceived health and perceived burden of the caregiver; and the functional capacity of the care receiver.

Perceived health was evaluated by the following question: “Would you say that your health status is very good, good, moderate, or poor?” For the different analyses, this variable was grouped into two categories: 1) very good/good and 2) moderate/poor.

The perceived burden was evaluated using the Burden Interview by Zarit et al. [4]. This scale was designed to assess the subjective overload experienced by caregivers of dementia patients and includes 22 items with responses on the following scale: 0 (never), 1 (rarely), 2 (sometimes), 3 (fairly often), and 4 (almost always). A higher score is related to a greater burden, and the total (summative) score ranges from 0 to 88 points. We used the Spanish version by Montorio et al. [38], who reported an internal consistency (Cronbach’s alpha) of 0.87 in the analyzed sample.

The functional capacity for BADL’s was evaluated using the 10-item Barthel Index [30] with a total score ranging from 0 to 100 (higher score = greater independence for BADL’s). We applied the version validated in Spain by Batzan et al. [39], who reported good psychometric properties (high criterion validity, 0.98 test-retest reliability, and 0.98 inter-observer reliability). In the analyzed sample, internal consistency (Cronbach’s alpha) was 0.92. In addition, we used the Barthel Index to rank the level of dependence of the car recipients according to the Shah et al.’s classification [40] (score of 0-20: total dependence, 21-60: severe dependence, 61-90: moderate dependence and 91-99: slight dependence).

### Procedure

Data were gathered through at-home interviews by a nurse trained for this purpose and with experience in this type of task. The nurse had no connections with the study participants.

After requesting the corresponding permissions, the case-management nurse of the Health Center was contacted to access the database of primary caregivers. The primary caregivers were contacted through their family nurse, who informed them about the study and asked their participation, and an appointment was arranged for an interview.

The study was approved by the Ethics Committee of our institution and followed the principles of the Helsinki Declaration [41]. Before interviews were conducted, participants were fully informed of the study objectives and their written consent was obtained. Participants were assured that their data would be treated anonymously and that their confidentiality would be guaranteed.

### Statistical analysis

Means with standard deviations and percentages were calculated. Correlation coefficients were determined in bivariate analyses, and multivariate analysis was conducted using multiple linear regression (stepwise method). The following assumptions were verified: (a) normality and homoscedasticity (residual plots), (b) linear relationship (partial regression plots), (c) independence of residuals (Durbin-Watson statistic), and (d) absence of collinearity (Tolerance and Variance Inflation Factor). P <0.05 was considered significant. SPSS v. 17.0 for Windows (IBM Inc., Chicago, IL, USA) was used for the statistical analysis.

## Results

The mean age of the caregivers was 61.7 yrs; 79.1% were female; 53.5% were the children of the care receivers, and 90.7% resided with them (Table 1). Regarding the level of dependence of the care recipients according to Shah et al.’s classification, 38.4% had total dependence, 45.6% severe dependence, and 16% moderate dependence.

**Table 1:** Description of the participants

		*M*	*SD*	Number	Percent
Age		61.7	13.6		
Sex	Female			68	79.1
	Male			18	20.9
Relationship	Spouse			31	36.0
	Daughter/Son			46	53.5
	Other			9	10.5
Co-residence	Yes			78	90.7
	No			8	9.3
Care duration (yrs)		9.5	11.8		

*M* mean, *SD* standard deviation

In the bivariate analyses (Table 2), the psychological dimension of quality of life was significantly associated with emotion-focused coping and with the age and perceived burden of the caregiver; the physical dimension was associated with age and perceived burden of the caregiver; the relational dimension was associated with perceived burden; and the environmental dimension was related to problem-focused, emotion-focused, and socially supported coping and to the perceived caregiver burden.

**Table 2:** Correlation matrix of the study variables

	1	2	3	4	5	6	7	8	9	10	11	12
1 Psychological QoL	1	0.68^a^	0.69^a^	0.62^a^	0.20	0.24^b^	-0.19	0.21	-0.23^b^	0.01	0.05	-0.56^a^
2 Physical QoL	0.68^a^	1	0.69^a^	0.64^a^	0.19	0.13	-0.06	0.10	-0.33^a^	0.01	0.01	-0.43^a^
3 Social rel. QoL	0.69^a^	0.69^a^	1	0.60^a^	0.20	0.16	-0.06	0.16	-0.19	0.14	-0.02	-0.38^a^
4 Environmental QoL	0.62^a^	0.64^a^	0.60^a^	1	0.24^b^	0.27^b^	-0.04	0.31^a^	-0.19	0.07	-0.06	-0.44^a^
5 Problem-focused coping	0.20	0.19	0.20	0.24^b^	1	0.71^a^	0.10	0.57^a^	-0.10	0.07	-0.09	-0.09
6 Emotion-focused coping	0.24^b^	0.13	0.16	0.27^b^	0.71^a^	1	0.19	0.49^a^	0.09	0.20	-0.05	-0.15
7 Dysfunctional coping	-0.19	-0.06	-0.06	-0.04	0.10	0.19	1	0.16	-0.05	0.04	-0.21	0.25^b^
8 Socially-supported coping	0.21	0.10	0.16	0.31^a^	0.57^a^	0.49^a^	0.16	1	-0.16	0.08	-0.17	0.07
9 Age (caregiver)	-0.23^b^	-0.33^a^	-0.19	-0.19	-0.10	0.09	-0.05	-0.16	1	-0.11	-0.06	-0.03
10 Sex (caregiver)	0.01	0.01	0.14	0.07	0.07	0.20	0.04	0.08	-0.11	1	0.15	0.11
11 Functional capacity	0.05	0.01	-0.02	-0.06	-0.09	-0.05	-0.21	-0.17	-0.06	0.15	1	0.03
12 Perceived burden	-0.56^a^	-0.43^a^	-0.38^a^	-0.44^a^	-0.09	-0.15	0.25^b^	0.07	-0.03	0.11	0.03	1
Possible range	7–35	5–25	3–15	6–30	12–60	20–100	16–80	12–60			0–100	0–88
Mean	18.4	20.9	9.0	26.1	30.9	50.6	28.7	29.4	61.7		39.9	33.3
Standard deviation	3.5	5.3	2.5	4.6	7.6	8.8	6.1	8.6	13.6		32.1	16

*Notes*: *QoL* quality of life

^a^Correlation is significant at 0.01 level (bilateral)

^b^Correlation is significant at 0.05 level (bilateral)

**Table 3:** Multiple linear regressions for the quality of life dimensions

Dependent variable	Independent variables	*B*	SE *B*	β	*p*	r^2^
Psychological dimension	Dysfunctional coping	-0.128	0.056	-0.277	0.028	0.49
	Emotion-focused coping	0.153	0.040	0.435	0.000	
	Perceived burden	-0.055	0.024	-0.277	0.029	
	Perceived health status of caregiver	10.839	0.730	0.283	0.015	
Physical dimension	Age	-0.153	0.050	-0.361	0.004	0.38
	Perceived burden	-0.098	0.042	-0.286	0.024	
	Perceived health status of caregiver	30.215	10.411	0.286	0.027	
Relational dimension	Age	-0.067	0.024	-0.338	0.007	0.35
	Female gender	10.716	0.655	0.313	0.012	
	Perceived burden	-0.062	0.019	-0.387	0.002	
Environmental dimension	Socially-supported coping	0.278	0.071	0.456	0.000	0.36
	Perceived burden	-0.121	0.037	-0.379	0.002	

Multiple linear regression models were constructed for each quality of life dimension (Table 3); independent variables in models were the four types of coping (problem-focused, emotion-focused, avoidance-type, and socially-supported) and the control variables (age, gender, perceived health status and burden of caregiver, and functional capacity of care receiver). Although high correlations were observed among problem-focused, emotion-focused, and socially-supported coping (Table 2), results of the multicollinearity diagnoses of the four models were acceptable, with tolerance values above 0.40; hence, all of these variables were considered in the models.

## Discussion

In this study of the primary caregivers of dependent elderly relatives, dysfunctional (passive or avoidance-type) coping was related to a worse quality of life in the psychological dimension, while emotion-focused and socially-supported coping strategies were associated with a superior quality of life in psychological and environmental dimensions, respectively. The physical and relational dimensions of quality of life were not related to the type of coping.

### Dysfunctional coping and quality of life

A relationship between dysfunctional coping and worse psychological dimension of quality of life has previously been reported in caregivers of the elderly with prostate cancer [18], the elderly with dementia [21], adults/elderly with epilepsy [23], and adults/elderly with traumatic brain injury [17]. The present findings strengthen available evidence on the relationship between avoidance-type coping and quality of life because of the adjustment for potential confounders. Dysfunctional coping has also been related to superior anxiety and depression in caregivers of the elderly with dementia [42]. The above results and our data support the hypothesis that avoidance-type coping impairs the health and quality of life of caregivers of the dependent elderly. Our findings, together with the results of previous studies, support that interventions aimed at decreasing dysfunctional coping could be helpful for the quality of life of caregivers caring for dependent elderly relatives.

### Active emotion-focused coping and quality of life

We could find no published studies on the relationship between quality of life and active emotion-focused coping as defined by Carver et al. [34]. Two studies [43, 44] reported a negative association between quality of life and emotion-focused coping, but this included both active emotion-focused coping and avoidance-type coping, which is known to be inversely related to quality of life, as noted above.

Our findings on emotion-focused coping and quality of life are consistent with previous reports on the relationship between active emotion-focused coping by caregivers and their improved emotional health. Various authors found that emotion-focused coping was negatively associated with anxiety and depression in caregivers of elderly people with dementia [42] and with anxiety in caregivers of elderly people with cancer [45]. The results for quality of life in our study, similar to those for anxiety and depression in the above studies, may be attributable to the greater effectiveness of active emotion-focused coping when the caregiver has a lower degree of control [46], as is often the case in care for dependent elderly relatives. As we have seen above, most of the second order dimensions in the Carver’s emotion-focused coping are based on the recognition, reappraisal, and acceptance of the caregiving situation. Thus, our findings support that interventions aimed at improving these recognition, reappraisal, and acceptance could be helpful for the quality of life of caregivers caring for dependent elderly relatives.

### Socially-supported coping and quality of life

Our results on socially-supported coping and quality of life differ from those of Yu et al. [22], who found no relationship between this coping strategy and emotional or physical quality of life. However, they studied only these two quality of life dimensions in comparison to the four dimensions considered in the present study, which may have masked the potential association with socially-supported coping. Our results also differ from those of Kate et al. [19] in schizophrenia caregivers. These authors found negative association between seeking social support and quality of life in the environmental dimension. However, no control for caregiver burden was performed, so that findings may be explained because more burdened caregivers seek more support. Our findings are consistent with the adaptive nature attributed to seeking social support coping [19] and support that interventions aimed to strengthen social support could improve the quality of life of caregivers of dependent elderly relatives.

### Problem-focused coping and quality of life

Our results on the lack of association between problem-focused coping and quality of life agree with the findings by Kershaw et al. [18] in caregivers of elderly prostate cancer patients and those by Chronister and Chan [17] in caregivers of traumatic brain injury patients. Our results are also consistent with the review by Li et al. [42], which found no association between problem-focused coping and anxiety or depression in caregivers of elderly relatives with dementia. These results and our data may reflect a greater effectiveness of problem-focused coping in situations when the degree of control is high but not when it is low [46], as in the care of a dependent elderly relative. Findings from Webb et al.’s study [47] are consistent with this argumentation. These authors found that problem-focused coping was related to a lower subjective burden in positive symptoms behavior of schizophrenia (perceived by caregivers as more solvable), whereas the same type of coping was related to a greater subjective burden in negative symptoms behavior (perceived by caregivers as less solvable).

### Limitations

Our study has some limitations. First, its descriptive cross-sectional design prevents the establishment of causal relationships, although the effect of coping on quality of life is supported by various conceptual models (e.g., [17]) and empirical results [17, 18]. Second, the study sample is non-probabilistic, although the risk of selection bias is reduced by the fact that participation was offered to all eligible subjects and only a small number refused participation. In addition, the study sample is very similar to that of the Spanish national cross-sectional survey for informal caregivers of older people [48] and can therefore be considered representative of Spanish informal caregivers.

## Conclusions

The following conclusions can be drawn from this study of the primary caregivers of dependent elderly relatives: 1) it is important to consider coping strategies in the assessment of primary caregivers of dependent elderly relatives; 2) the quality of life of caregivers is related to their coping strategies, 3) their quality of life can be worsened by avoidance-type coping, and 4) their quality of life can be improved by active emotion-focused coping and socially-supported coping.

### Relevance to clinical practice

Within the limitations of a cross-sectional study, these conclusions may be useful for the development of interventions in caregivers of dependent elderly relatives to reduce avoidance-type coping and favor acceptance of their situation and the search for social support. Among these interventions, we have problem-solving interventions [49], benefit-finding and positive reappraisal interventions [50], acceptance and control of dysfunctional thoughts [51], and interventions aimed to strengthen social support [52].

## Abbreviation

BADL: Basic activity of daily living

## References

[1] OECD (2013). Health at a Glance 2013. OECD indicators.

[2] Pinquart M, Sorensen S (2003). Differences between caregivers and noncaregivers in psychological health and physical health: A meta-analysis. Psychol Aging.

[3] Lazarus RS, Folkman S (1984). Stress, appraisal and coping.

[4] Zarit SH, Reever KE, Bach-Peterson J (1980). Relatives of the impaired elderly: correlates of feelings of burden. Gerontologist.

[5] Gräßel E, Adabbo R (2011). Perceived burden of informal caregivers of a chronically ill older family member: Burden in the context of the transactional stress model of Lazarus and Folkman. GeroPsych (Bern).

[6] Kinsella G, Cooper B, Picton C, Murtagh D (1998). A review of the measurement of caregiver and family burden in palliative care. J Palliat Care.

[7] Pearlin LI, Mullan JT, Semple SJ, Skaff MM (1990). Caregiving and the stress process: an overview of concepts and their measures. Gerontologist.

[8] Del-Pino-Casado R, Frias-Osuna A, Palomino-Moral PA, Pancorbo-Hidalgo PL (2011). Coping and subjective burden in caregivers of older relatives: a quantitative systematic review. J Adv Nurs.

[9] Moos RH, Brennan PL, Fondacaro MR, Moos BS (1990). Approach and avoidance coping responses among older problem and nonproblem drinkers. Psychol Aging.

[10] Penley JA, Tomaka J, Wiebe JS (2002). The association of coping to physical and psychological health outcomes: a meta-analytic review. J Behav Med.

[11] Haley WE, Levine EG, Brown SL, Bartolucci AA (1987). Stress, appraisal, coping, and social support as predictors of adaptational outcome among dementia caregivers. Psychol Aging.

[12] Pruchno RA, Resch NL (1989). Mental health of caregiving spouses: coping as mediator, moderator, or main effect?. Psychol Aging.

[13] Bass D (2002). Content and implementation of a caregiver assessment. National Family Caregiver Support Program Issue Brief.

[14] Folkman S (2008). The case for positive emotions in the stress process. Anxiety Stress Coping.

[15] Greenglass ER, F E (2002). Proactive coping and quality of life management. Beyond coping: Meeting goals, vision, and challenges.

[16] WHOQoL Group (1993). Study protocol for the World Health Organization project to develop a Quality of Life assessment instrument (WHOQOL). Qual Life Res.

[17] Chronister J, Chan F (2006). A stress process model of caregiving for individuals with traumatic brain injury. Rehabil Psychol.

[18] Kershaw TS, Mood DW, Newth G, Ronis DL, Sanda MG, Vaishampayan U, Northouse LL (2008). Longitudinal analysis of a model to predict quality of life in prostate cancer patients and their spouses. Ann Behav Med.

[19] Kate N, Grover S, Kulhara P, Nehra R (2014). Relationship of quality of life with coping and burden in primary caregivers of patients with schizophrenia. Int J Soc Psychiatry.

[20] Ekwall AK, Sivberg B, Hallberg IR (2007). Older caregivers’ coping strategies and sense of coherence in relation to quality of life. J Adv Nurs.

[21] Riedijk SR, De Vugt ME, Duivenvoorden HJ, Niermeijer MF, Van Swieten JC, Verhey FR, Tibben A (2006). Caregiver burden, health-related quality of life and coping in dementia caregivers: a comparison of frontotemporal dementia and Alzheimer’s disease. Dement Geriatr Cogn Disord.

[22] Yu Y, Hu J, Efird JT, McCoy TP (2013). Social support, coping strategies and health-related quality of life among primary caregivers of stroke survivors in China. J Clin Nurs.

[23] van Andel J, Westerhuis W, Zijlmans M, Fischer K, Leijten FS (2011). Coping style and health-related quality of life in caregivers of epilepsy patients. J Neurol.

[24] Helder DI, Kaptein AA, Van Kempen GM, Weinman J, Van Houwelingen JC, Roos RA (2002). Living with Huntington’s disease: illness perceptions, coping mechanisms, and spouses’ quality of life. Int J Behav Med.

[25] Yang X, Hao Y, George SM, Wang L (2012). Factors associated with health-related quality of life among Chinese caregivers of the older adults living in the community: a cross-sectional study. Health Qual Life Outcomes.

[26] McCullagh E, Brigstocke G, Donaldson N, Kalra L (2005). Determinants of caregiving burden and quality of life in caregivers of stroke patients. Stroke.

[27] Chronister J, Chan F, Sasson-Gelman EJ, Chiu CY (2010). The association of stress-coping variables to quality of life among caregivers of individuals with traumatic brain injury. NeuroRehabilitation.

[28] White CL, Mayo N, Hanley JA, Wood-Dauphinee S (2003). Evolution of the caregiving experience in the initial 2 years following stroke. Res Nurs Health.

[29] Myaskovsky L, Dew MA, Switzer GE, McNulty ML, DiMartini AF, McCurry KR (2005). Quality of life and coping strategies among lung transplant candidates and their family caregivers. Soc Sci Med.

[30] Mahoney F, Barthel D (1965). Functional evaluation: The Barthel Index. Md State Med J.

[31] Nunnally J, Bernstein I (1994). Psychometric theory.

[32] Cohen J (1988). Statistical power analysis for the behavioral sciences.

[33] Crespo M, Cruzado J (1997). La evaluación del afrontamiento: adaptación española del cuestionario COPE con una muestra de estudiantes universitarios. Anál Modif Conducta.

[34] Carver CS, Scheier MF, Weintraub JK (1989). Assessing coping strategies: a theoretically based approach. J Pers Soc Psychol.

[35] Litman JA (2006). The COPE inventory: dimensionality and relationships with approach-and avoidance-motives and positive and negative traits. Pers Individ Dif.

[36] The WHOQOL Group (1998). Development of the World Health Organization WHOQOL-BREF quality of life assessment. The WHOQOL Group. Psychol Med.

[37] Badia X, Salamero M, Alonso J (2002). La medida de la salud: Guía de escalas de medición en español [The measure of Health: guide for scales in Spanish].

[38] Montorio I, Izal M, López A, Sánchez M (1998). La entrevista de carga del cuidador. Utilidad y validez del concepto de carga. Anal Psicol.

[39] Baztán JJ, Pérez J, Alarcón T, San Cristóbal E, Izquierdo G, Manzarbeitia I (1993). Indice de Barthel: Instrumento válido para la valoración funcional de pacientes con enfermedad cerebrovascular [Barthel Index: A valid tool for functional assessment in stroke patients]. Rev Esp Geriatr Gerontol.

[40] Shah S, Vanclay F, Cooper B (1989). Improving the sensitivity of the Barthel Index for stroke rehabilitation. J Clin Epidemiol.

[41] Declaration of Helsinki - Ethical Principles for Medical Research Involving Human Subjects. http://www.wma.net/en/30publications/10policies/b3/. Accessed 12 Jan 2016.

[42] Li R, Cooper C, Bradley J, Shulman A, Livingston G (2012). Coping strategies and psychological morbidity in family carers of people with dementia: A systematic review and meta-analysis. J Affect Disord.

[43] Green MR. Coping and mental health among patients with end-stage pulmonary disease and primarycaregivers. Doctoral dissertation. Ohio State University, Psychology. Columbus: Ohio State University; 2009.

[44] Klum MA. Coping in caregivers of family members with traumatic brain injury and the effects on the caregivers' quality of life. Doctoral dissertation. Massey University, Psychology. Albany: Massey University; 2012.

[45] Goldzweig G, Merims S, Ganon R, Peretz T, Baider L (2012). Coping and distress among spouse caregivers to older patients with cancer: An intricate path. J Geriatr Oncol.

[46] Wartella JE, Auerbach SM, Ward KR (2009). Emotional distress, coping and adjustment in family members of neuroscience intensive care unit patients. J Psychosom Res.

[47] Webb C, Pfeiffer M, Mueser KT, Gladis M, Mensch E, DeGirolamo J, Levinson DF (1998). Burden and well-being of caregivers for the severely mentally ill: the role of coping style and social support. Schizophr Res.

[48] Instituto de Mayores y Servicios Sociales (2005). Cuidados a las personas mayores en los hogares españoles. El entorno familiar [Care for elder people in Spanish homes. Family environment].

[49] Meyers FJ, Carducci M, Loscalzo MJ, Linder J, Greasby T, Beckett LA (2011). Effects of a problem-solving intervention (COPE) on quality of life for patients with advanced cancer on clinical trials and their caregivers: simultaneous care educational intervention (SCEI): linking palliation and clinical trials. J Palliat Med.

[50] Cheng ST, Lau RW, Mak EP, Ng NS, Lam LC (2014). Benefit-finding intervention for Alzheimer caregivers: conceptual framework, implementation issues, and preliminary efficacy. Gerontologist.

[51] Losada A, Marquez-Gonzalez M, Romero-Moreno R, Mausbach BT, Lopez J, Fernandez-Fernandez V, Nogales-Gonzalez C (2015). Cognitive-behavioral therapy (CBT) versus acceptance and commitment therapy (ACT) for dementia family caregivers with significant depressive symptoms: Results of a randomized clinical trial. J Consult Clin Psychol.

[52] Mittelman MS, Roth DL, Haley WE, Zarit SH (2004). Effects of a caregiver intervention on negative caregiver appraisals of behavior problems in patients with Alzheimer’s disease: results of a randomized trial. J Gerontol B Psychol Sci Soc Sci.

